# A Review of J. C. Reeve, M. D., on "Anæsthetics in Dental Practice."

**Published:** 1888-12

**Authors:** S. J. Hayes

**Affiliations:** Pittsburg, Pa.


AMERICAN JOURNAL

—OF—

DENTAL SCIENCE.

Vol. xxii. Third Series—DECEMBER, 1888. No. 8.

ARTICLE I.

### A REVIEW OF J. C. REEVE, M. D., OF DAYTON, O., ON "ANÆSTHETICS IN DENTAL PRACTICE."

BY S. J. HAYES, PITTSBURG, PA.

My attention has just been called to an article that appears in the October number of your journal in which some very *unjust* as well as *unkind* remarks in reference to the *knowledge* and qualifications of dentists in the administration of anæsthetics, etc., are made, to which (through your kind indulgence) I desire to refer and if possible disabuse the author's mind of some of the errors he has espoused and is *tenaciously advocating*.

I might not feel disposed to take special notice of the article in question, were it not that the author is quite prominent as a surgeon, stands at the head of a hospital staff in his city, is somewhat "looked up to" for the knowledge that he *assumes* to possess and seems to believe that what he does not know about anæsthetics and anæsthesia is not worth knowing. In fact, he assumes to be an educator of the dental profession and one of high authority in the article to which we now refer. It is *flauntingly headed*

"ANÆSTHETICS IN DENTAL PRACTICE."

BY J. C. REEVE, M. D., DAYTON, OHIO.

Thus it will be seen that he feels the importance of his *superior knowledge* of the science of anæsthesia and anæsthetics as well as such a deep interest in the welfare of humanity and *especially* of the *dental profession* that he *magnanimously* volunteers to give the dental profession the benefit of his superior knowledge and extensive experience as well as *sound advice* to leave anæsthetics alone and turn the use of them over to the M. D's.

But as we find even in the present age of progression, too many in both the dental and medical professions and *especially* in the *latter*, who are quite thoroughly educated without being learned, or possessing very little learning or knowledge, it is *important* that we discriminate in the investigation of science between the mere *opinions*, or the *dogmas* of those who *assume* to be our educators and guardians and the truths pertaining to the science. Some men possess a *jealousy* of the knowledge of others and of other professions of a kindred nature without being conscious of the fact. I will not say that this seems to be pre-eminently the case with Dr. Reeve as I might be liable to the charge of doing him injustice, but will allow his own language in his premises to represent him—"As I am *decidedly opposed* to the administration of chloroform and ether by dentists, I will commence by disclaiming any disposition to consider the paper or the subject in any other spirit than that of science." The author is here referring to an article that appears in the September number of the *Dental Register* from the pen of J. E. Harris, M. D., D. D. S., but as he dwells in his remarks very little upon the article, we shall only deem it our duty to treat of the thoughts that he advances in his article, under the influence of the *genial rays* of the science to which he is so devoted.

As the doctor refuses to "consider the subject in *any other spirit* than that of science" it may not be amiss to remind our *learned* friend that "science embraces those branches of knowledge which give a positive statement of truths as found in the nature of things," and that when we investigate science, we are after truths and not mere *opinions* or *dogmas.*

Now, I propose to show that this self-constituted educator of the dental profession and gracious benefactor of the public, like many other M. D's who assume to *know* and to have *superior knowledge,* is *utterly ignorant* of the first principles or A. B. C. of the science to the defence of which he has devoted the article in question. In so doing, I shall not call in question his success as a surgeon, as that does not depend so much upon *knowledge* of any science as upon *self-confidence* and *assurance,* of which the author—it will be readily seen—has an abundance. But I will simply demonstrate that Dr. Reeve displays less knowledge of the science of anæsthesia and anæsthetics than would be expected of the most *ignorant charlatan,* though he may have used the agencies to which he refers extensively.

In the first place, the M. D. does not appear to have a clear conception or knowledge of what constitutes anasthesia, and consequently, is unable to distinguish between an anæsthetic and an asphyxiating agent and hence (I submit), is utterly incompetent either to *select* or *administer* an anæsthetic. For if a certain or definite condition be necessary for a *safe* and *painless* surgical operation, it is *very important* that the person who undertakes to put the patient in that condition, should know *definitely* what that condition is, as well as to know in what respect it differs from other conditions to which it bears some resemblance, but which are known to be *injurious* and *dangerous.*

It *is*, and *must* be admitted that this very desirable condition is the one in which the *forces* and *functions* of life, are in continuance under modification. This modification may be induced by suitable narcotics or by *free oxygen* or by both whereby an absence of sensation is attained. The *force* of life or that out of which *vital force*, is generated, is free oxygen. This is found abundantly in the atmosphere, and is in fact the only element undcr heaven that will support combustion or sustain either vegetable or animal life. Therefore, it is the *all important* element in your anæsthetic and without which you cannot have an anæsthetic nor can you produce anæsthesia. Any agency that does not contain *free oxygen* when administered, can only produce asphyxia and if continued death. It does not matter what your agency is, if it dces not have sufficient *free oxygen* to sustain life it cannot produce anæsthesia, but will inevitably produce asphyxia and if continuously administered death. And yet, Dr. Reeve calls nitrous oxide gas an anæsthetic and commends it to the use of the dental profession; when if he knows anything about its composition, he should know that it has not a particle of *free oxygen* in it and that it can only produce asphyxia which always does such *violence* to the *laws* of nature no matter whether it be produced under water, in the *mines*, or by giving exclusively nitrous oxide gas, olyphiant gas, carbonic acid gas, nitrogen gas, or chloroform or ether or anything else that does not contain sufficient *free oxygen* to sustain life, that *great injury* to the *nervous* and *physical* constitution must *inevitably* be the result. Therefore, as the eminent Dr. Richardson said in one of his lectures "the use of nitrous oxide gas should be *prohibited* by law." While *insensibility* is an attribute of asphyxia as well as of anæsthesia, yet they are *radically* different and *diametrically* opposite in their tendencies and results. The former may be properly designated as "*the first step in at death's door*," while the latter is the happy state of *insensibility* with the continuance of the forces and functions of life under modification. If chloroform, ether, nitrous oxide gas or anything else, that does not contain *free oxygen* be given without admitting the air by which *free oxygen* is supplied, nothing but *asphyxia* and *death* can be the result. Hence, he who cannot recognize the radical difference between the conditions known *properly* as anæsthesia, syncope and paralysis, should not be permitted to administer an anæsthetic. no matter whether he be an M. D., or a D. D. S. For neither the M. D., nor the D. D. S. appended to a man's name, makes him of itself any more competent to select or administer an anæsthetic, only as it may exist in the minds of those of very *limited* knowledge who have been educated to think that the M. D. has a magic that *transforms* and confers *omnicience* upon the otherwise ordinary personage.

But why is Dr. Reeve so "decidedly opposed to the administration of such anæsthetics as chloroform and ether by dentists?" Is it because they have less acquaintance with the so-called anæsthetics or know less of the science of anæsthesia than the physicians? To which profession is the credit due for the discovery and introduction of the most of the pain obtunding agencies that are used to-day? Dr. W. Wells, a well-known dentist, who discovered that insensibility to pain could be produced by the use of *nitrous oxide* gas, was the first to give to the notion its practical embodiment by the extraction of a tooth without pain. Dr. Morton, a former pupil of Dr. Wells, who discovered that ærated ether could produce anæsthesia, was the first to introduce its use as a pain obtunding agency in the painless extraction of teeth. For tooth-drawing chloric ether was first employed in this country by Dr. Jacob Bell, and Dr. Sansom, an English writer says: (page 259 "Sansom on Chloroform.") "Since the introduction of chloroform that agent has been employed almost *exclusively* in this country as an anæsthetic in dental operations." But when these agencies were first introduced a terrible *remonstrance* was presented by many of the M. D'.s at the use of these dangerous elements and then as now they were the accusers of the dental profession for the using of agencies of which they know nothing about, thus pretending that they know all about them and that they are the proper custodians of humanity.

As Dr. J. E. Harris, in the paper on which Dr. Reeve bases some of his remarks, properly and *correctly*, intimates that the main reason why the *general impression* is extant that "it is more dangerous to administer anæsthetics for dental operations than for general surgery" and that more deaths have occurred in the dental chair than on the gynæcological chair or table, "is the *great difference* in the *sequel* to a death under the hands of a general surgeon and a death under the hands of a dentist or oral surgeon." If a physician loses a patient from the use of the so-called anæsthetics, there is first usually a better opportunity to *cover up* or *conceal* the real cause of the death and no doubt thousands af deaths, that have been caused by the *improper* preparation and administration of anæsthetics and were in *reality* deaths from *mal-practice*, have never been reported. The cause of death having been assigned to the nature of the *severe* operation, or to the peculiar diseased condition of the patient. If the death occurs during the administration of the anæsthetic and previous to commencing the surgical operation, there is *usually* an effort to find a diseased condition of the heart, the lungs, or the brain, and of course that is not difficult to find and the physician is *relieved* of a suit at court for *mal-practice* and the *odium* that might otherwise rest upon him in his community. He has several influential friends in his own profession and usually of his own school (physicians are not generally jealous of each other as dentists are of their brethren,) who sympathize with him and are anxious to come to his rescue. He suggests to them that an *autopsy* be held with the consent of the friends of the deceased and that *they* should conduct the same and that *they* can either find clots of blood in the heart and assign that as an evidence of valvular disease of that organ, or find the lungs diseased or a congested condition of the brain, the arteries contracted and evidence of *embolism* and announces the *one* or the *other* of these as the cause of the death. Yet, who does not know that even a dead ox will have clots of blood in the heart, and that even alcohol much more so ether or chloroform, will produce a congested condition of the brain, contract the arteries and cause evidence of embolism.

But if a death occurs in a dental chair during the administration of an anæsthetic and operation, it is difficult create the impression that the natnre of the operation was such that the patient might have died any hour, that the disease was of such a severe nature that the patient died from the effects of the surgery. Then again—and it is to be regretted—his brother dentists don't feel the necessity of coming to his rescue. In fact, many of them would rather see him suffer as that might slightly increase their practice and yearly income. And the physicians too often say he had *no business* to give the anæsthetic. He should have called in a physician. No autopsy is held, and the dentist, no matter how much better qualified he may be to administer an anæsthetic than the physician, is left alone to bear the burden of the *unfortunate* occurrence. No man, either in the dental or medical profession, who has been an observer of these incidents but that knows that I am relating the facts. The curse of this *wide-spread impression* not only lays at the door of the medical profession but also, with the dental profession, and the time has come when this fatal *error must* be corrected and the *odium wiped* from the garments of the dental profession. In order to *verify* these statements I may be pardoned for an allusion to personal observations, experience and knowledge of both the medical and dental professions as to their qualifications respectively to the successful use of anæsthetics and the so-called anæsthetics. During the past two years, I have had the pleasure of talking to thousands in both professions at the medical and dental colleges and societies, upon the science of anæsthesia and anæsthetics, and have given over fifteen hundred clinics in the use of "Hayes' Process of, and Apparatus for Generating and Applying Anæsthetics," etc., which has afforded an opportunity of acquiring a knowledge of the attainments, experience and qualifications of men in both the professions as pertaining to the science, and use of anæsthetics. While I have found many in both professions who have extensively read and studied the science and have had a great deal of experience in the use of pain obtunding agencies; yet, I have ascertained that there is no other science pertaining to the healing art, that is so little understood as the science of anæsthesia, and (I regret to say) especially in the medical profession. Though there are only about 20,000 dentists in the United States to 100,000 physicians yet, the dental profession have occasion to administer anæsthetics about ten times to the medical profession once, and as a consequence the dentists are *pressed into the service* and become more deeply interested. They are *forced* to read up and study the science of anæsthesia and the effects of the agencies employed. While very many of the physicians administer on an average scarcely once a month or once a year and some not at all. True, many of the surgeons in the hospitals and colleges and a few outside, have quite an extensive experience in the use of anæsthetics; but many of these even have given very little attention to *cause* and *effect* or the *fundamental* principles of the science. Others by the careful study of *cause* and *effect*, and skill in diagnosing conditions and causes of conditions, have become quite successful in the production of anæsthesia, but these are *exceptions* to the general run of the medical practitioners. On the other hand, it is *quite different* with men in the dental profession. You cannot find a dentist but that has occasion to use anæsthetics. There is a very *great demand* and he must supply the demand or lose a portion of his legitimate practice. Therefore, he eagerly casts about for the *best*, *safest* and most convenient agency to produce insensibility to pain, This accounts in part at least for the dentists having discovered and brought into use nearly all the pain obtunding agencies that are in use at the present time. Yet, in the face of these facts listen to the *preposterous* declaration of Dr. Reeve: "Certainly anæsthetics are administered for general surgical purposes hundreds of times for once in dental practice, and if so then the relative number of deaths under tooth-drawing is enormously large." Is there another physician in all this *enlightened* land that could be induced to make a similar declaration? To put the most charitable construction upon this *wild dogmatic assertion*, it must be presumed that the doctor's acquaintance with the relative duties of both the professions must be very limited indeed.

Again, in proof that "anæsthetics are more dangerous in dental practice than in surgery" he refers to the statistic reports of the relative causes of deaths as pertains to the nature of the operations from the use of chloroform in anæsthesia, as given by Dr. Sansom and by the Committee of the Royal Medico-Chirurgical Society. But even this the Doctor fails to report accurately. Instead of eight cases of death when teeth were either removed or to be removed out of 107 deaths of all surgical operations, he gives eight cases of death out of 100 as reported by Dr. Sansom, and instead of twelve cases of death from anæsthesia while teeth were extracted out of 109 deaths from all surgical operations, he gives twelve cases of death out of 107 as given by the Committee of R. M. S., yet, these reports instead of *substantiating* the Doctor's extravagant statement only *condemn* the same. It will be borne in mind that the reports only give a certain number of administrations out of which so many deaths occurred. It is certainly a very low estimate to place the half of those administrations for the painless extraction of teeth which should give the half of the mortality in either report, but instead of half the mortality, we have about one tenth laid at the door of the dental profession and nine-tenths at the door of the medical profession. Therefore, from these or any other reports, it is *not true* that more deaths have occurred in the dental chair, than at the hands of the general surgeons. But it *is trne*, that when a death does occur in the dental chair, instead of a *post-mortem* examination and an effort to cover up the real cause of the death, as is the case usually when the death occurs at the hands of the general surgeon; the coroner is called in to investigate the cause and testimony of so-called experts, usually from the medical profession, is heard from the *witness stand*, who are there not in *defense* of the dentist as they from their standpoint usually have some misgivings and distrust toward the dentist as he *might have called* them in to do this work; but they are there summoned by the coroner to assist him in pointing out the real cause of the death and they *seldom fail* to put it where it belongs. Then the report is published in all the local and distant secular papers and the medical and dental journals pick it up from these with their constructions and comments. But how different when a death occurs in general surgery. The *autopsy*, the medical society, the medical and dental journals, all favor the *unfortunate* physician. Usually it is quieted and does not reach the journals, frequently does not even get into the local medical society. Thus thousands of deaths, no doubt, caused from the *miserable* manner of *preparing* and administering anæsthetics, have never been reported and do not appear in the mortality reports from the use of anæsthetics. Now, I submit, whether this is justice alike to both professions and whether it is not *high time* that a *new leaf* be turned and that the dental profession shall demand the same protection from society and the respective professions as is accorded to the medical profession. The dentist of to-day is not a gunsmith, a blacksmith, or a barber, but has the honor of belonging to the *most progressive profession* of the age. His education and experience in special surgery and knowledge of the science of anæsthesia and experience in the use of anæsthetics entitle him to equal rank with the M. D., equal honor and protection from society and the *laws* of our country. In fact many physicians recognize the worth of the dentist's qualifications in the administration of anæsthetics and in *critical cases* prefer the assistance of their dentist in the use of anæsthetics to that of the medical practitioner notwithstanding the earnest protests of our noted surgeon of Dayton. In still further proof of these facts, may I be permitted to state that during the past ten years in the line of my duties with " Hayes Process of, and Apparatus for Generating and Applying Anæsthetics," I have administered chiefly for the extraction of teeth to nearly 9000 persons, without causing a death, or producing paralysis of the functions of life, or *asphyxia*, and with but a *very few cases* of nausea. Moreover, from the many who are now using this process, apparatus, etc., (mostly dentists) it would be a low estimate to put the number of administrations by dentists at 60,000, without having a single death during the administration or operation. I refer to the number of administrations without causing a death not only to *verify these* statements but to disprove another *absurd* statement which, however, the Doctor is not alone in making, namely, that in consequence of " the particular nerve involved in the dental operation, the acute pain caused by injuries to it and the powerful effect of sudden impressions upon its branches, upon the great and vital processes of respiration and circulation, by sudden impressions upon this nerve more than any other is that inhibition of the heart's action brought about, which is sudden death." And that as " anæsthesia for tooth-drawing is likely to be incomplete and will pretty certainly be so, if the operator is also the administrator," and that a "state of partial anæsthesia is therefore one of special danger and especially so if the pain produced is at once sudden and sharp," and that " such reflex actions are increased under chloroform."

Now, I admit that the anæsthesia is not usually as profound, *nor should it be*, for the removal of teeth as for most other surgical operations, and for this reason : that if the patient loses the power to control the muscles pertaining to the oral cavity, and a tooth or a root should happen to slip from the forceps, which will occur sometimes in the hands of the most skillful, it is liable to enter the œsophagus or larynx and cause trouble. But, if the patient be allowed to control these muscles though he may be unconscious and insensible to pain, he will exclude the tooth. But, I deny *most emphatically* that the reflex actions are increased under partial chloroform narcosis. This is *most certainly* one of the most misleading fallacies taught. Does chloroform or any other narcotic make sensation more acute? Is it not a fundamental law of anæsthesia that sensation diminishes as narcosis increases? And that the diminution of sensation does not *increase* vasco-motor activity, but on the contrary has a tendency to retard? The inhibition of the heart's action can only be brought about by the suspension of the circulation of *nervo-vital fluid*, or animo-electricity in the inhibitory nerve, as that is the motive power of the heart's action. This suspension can only be produced by concussion and revultion, the result of which is the *cessation* of the functions of life. This concussion or sudden arrestation of nerve circulation, may be produced by a sudden action of the intellect as by fright or the sudden breaking of very sad news, or it may be produced by an external agent such as the sudden introduction of an over volume of electricity or by sudden production of very severe pain, or a sudden introduction of an over percentage of a powerful narcotic. But, if sensation diminishes as narcosis increases, there certainly will be *less severe* and *less sudden* pain in partial anæsthesia than in case of no anæsthesia. In other words, there must be less liability to concussion or shock in partial narcosis than when chloroform has not been given at all. This is *inevitable*, and forcibly demonstrates the *fallacy* of the theory advanced by such theorists. Therefore, partial anæsthesia by the use of ærated chloroform, ærated ether or any other suitable narcotic preparation, does not *enhance* the dangers to life from the operation, but on the contrary diminishes the dangers. However, if the administrator of the anæsthetic neglects to describe its sensations in the periphery, in the fingers, hands, knees and body, the patient may mistake these for the approach of death and be so frightened as to produce *concussion*, or sudden *arrestation* of the circulation of animo-electricity or nervo-vital fluid in the inhibitory nerve and death may be the result. But the result is not due to the partial narcosis but mainly to the neglect of the administrator and more directly to the fright. Yet in complete or profound anæsthesia the patient passes through the very *same experience* and therefore partial anæsthesia cannot be more dangerous from that cause than *profound* anæsthesia and there must be less danger from the operation with partial narcosis than without any narcosis.

Again, we have only to call attention to Dr. Reeve's own language to show that he does not understand or know what he is talking about. He says: " Far more important than all, however, is the fact that the *induction* of *anæsthesia* for tooth-drawing is likely to be incomplete." Does the Doctor not know that the word anæsthesia represents condition? *Anæsthetics* are *inducted* to produce the condition known as Anæsthesia. This is not a *lapsus lingua* or slip of the pen, but a deliberate statement from an assumed educator of the dental profession. Other statements *equally intelligent* may receive attention in a future article, as well as the paper to which the Doctor refers in his *caution* against the use of ether by the dental profession.

